# Supplementary material for: Genomic Insight into Primary Adaptation of Mycobacterium tuberculosis to Aroylhydrazones and Nitrofuroylamides In Vitro
Source: Antibiotics (Basel). 2025 Feb 22;14(3):225. doi: 10.3390/antibiotics14030225 (PMC11939388; doi:10.3390/antibiotics14030225)
Supplement: Supplementary file 1 [file antibiotics-14-00225-s001.zip › Figures S1-S5.pdf]

Genomic Insight into Primary Adaptation of *Mycobacterium tuberculosis* to Aroylhydrazones and Nitrofuroylamides *in vitro*

Igor Mokrousov, Violina T. Angelova, Ivaylo Slavchev, Mikhail Bezruchko, Simeon Dimitrov, Dmitrii E. Polev, Georgi M. Dobrikov, Violeta Valcheva

Supplementary Figures S1-S5. Gene-gene networks of genes *Rv3755c*, *ppgK*, *mmpS2*, *infB*, *nrdH*

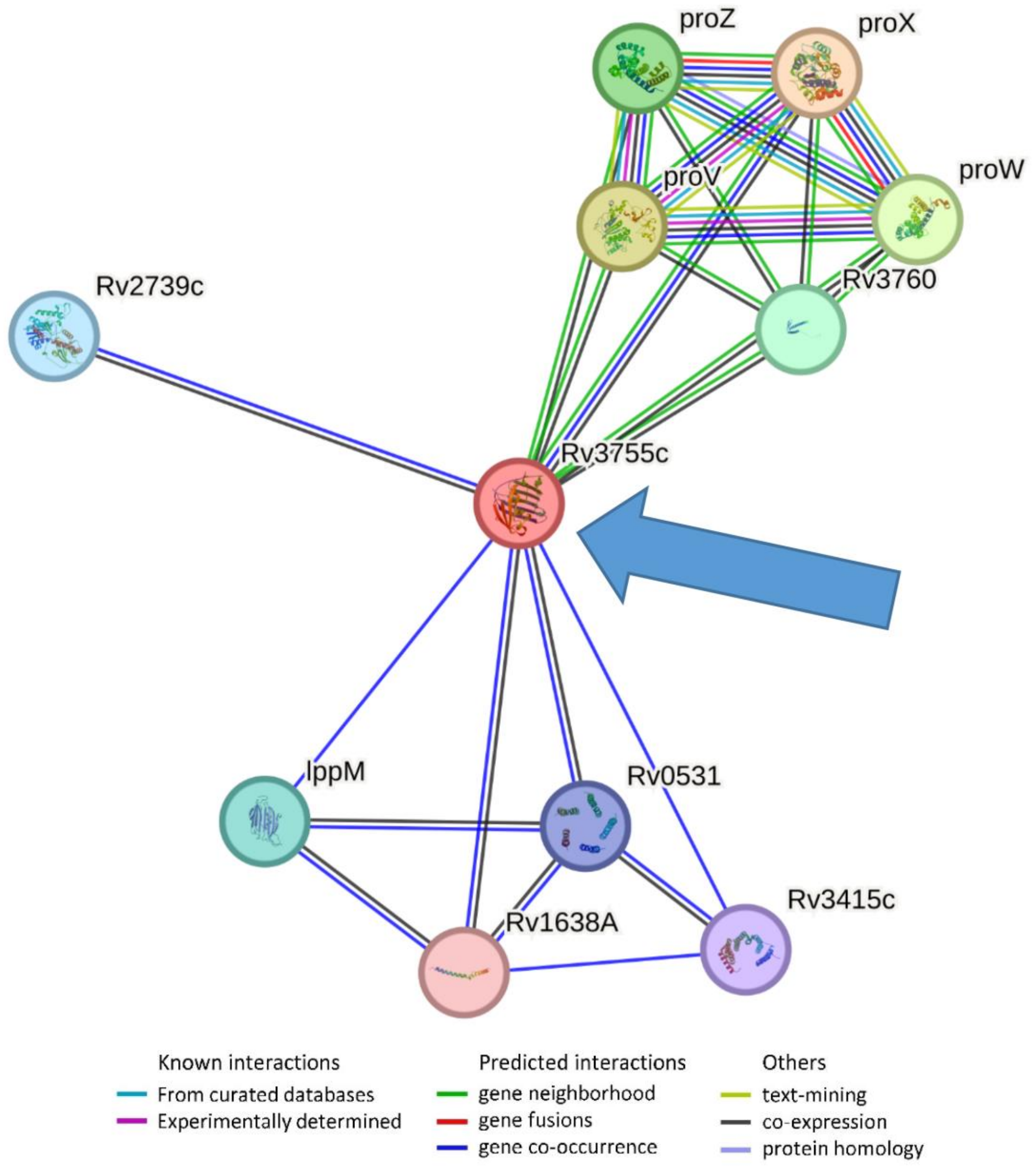

Figure S1. *Rv3755c* gene-gene network.

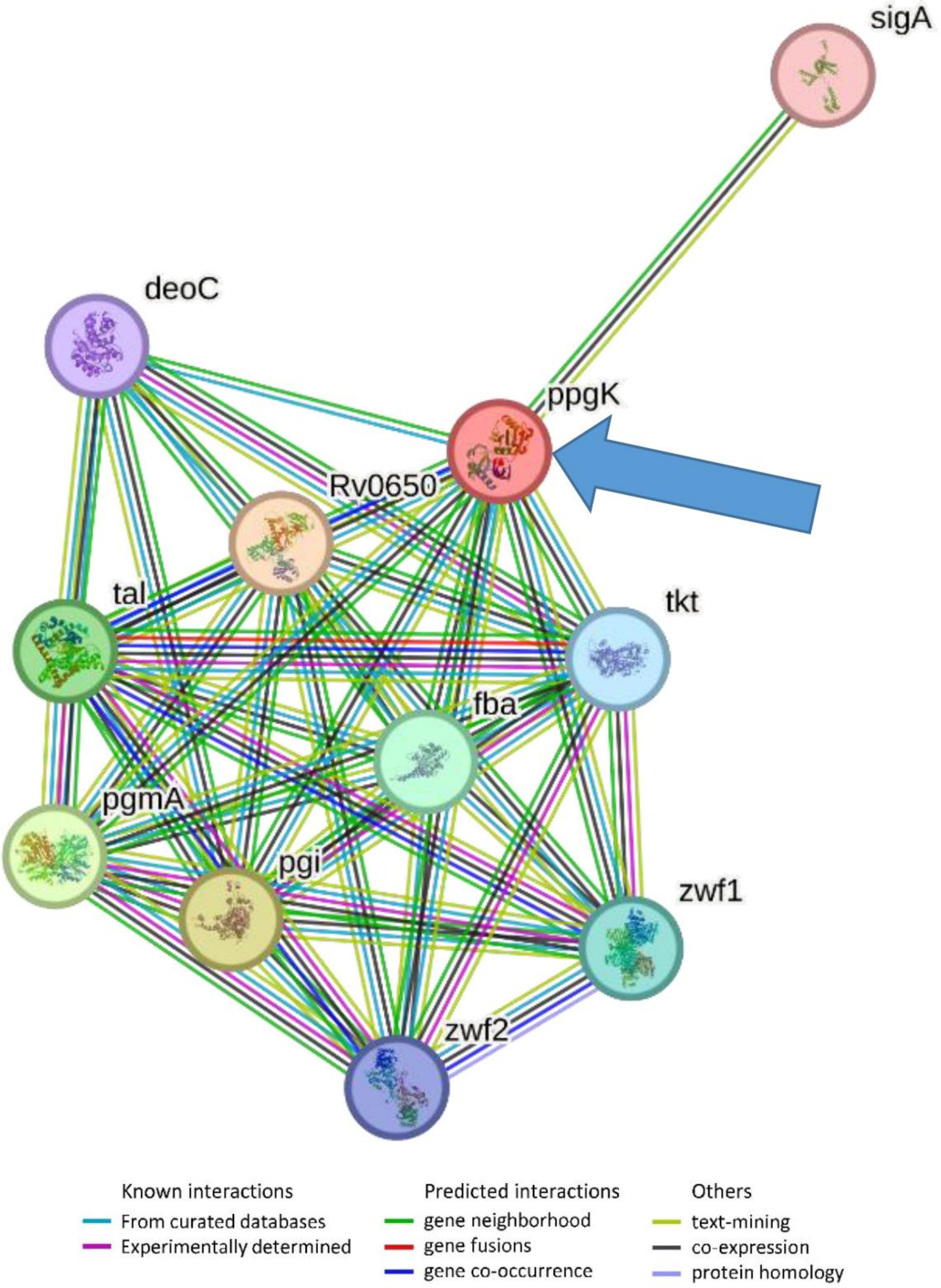

Figure S2. *ppgK* gene-gene network. *ppgK* is shown by an arrow.

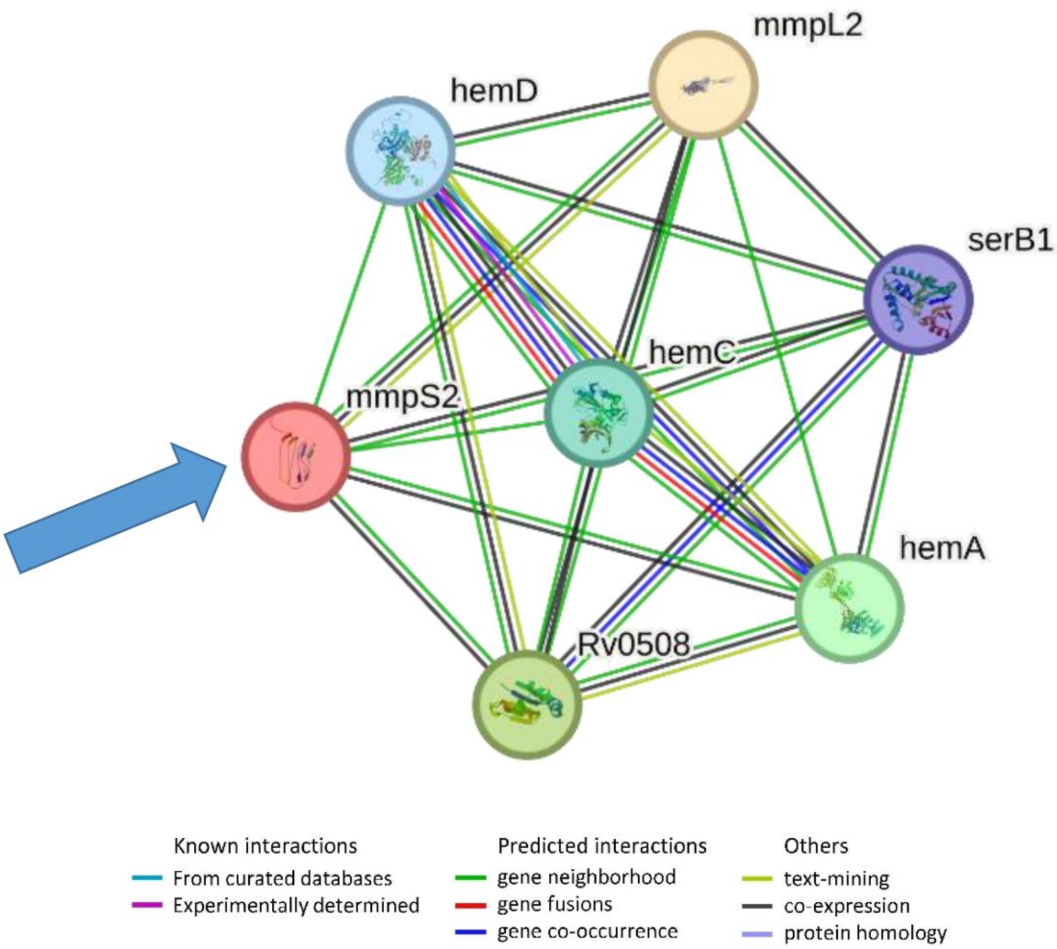

Figure S3. *mmpS2* gene-gene network. *mmpS2* is shown by an arrow.

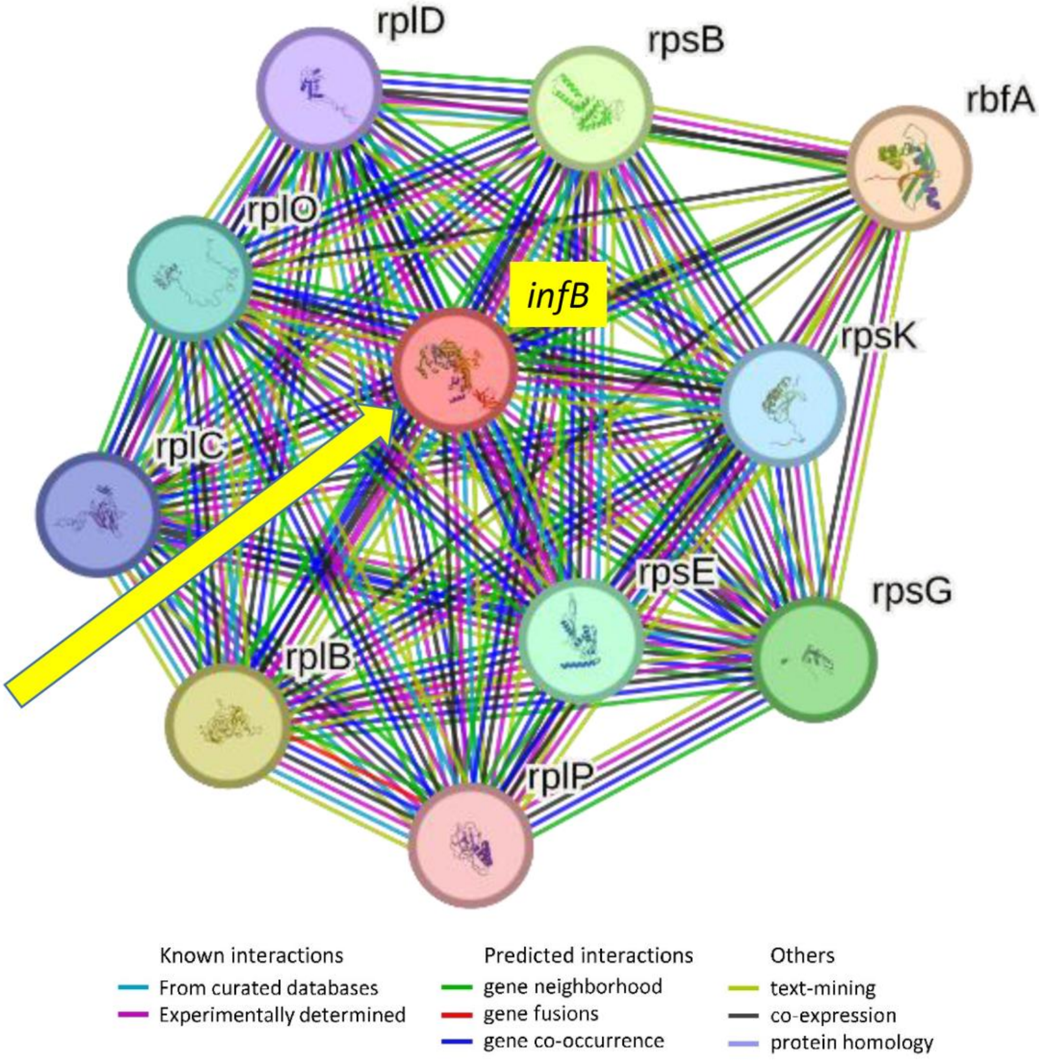

Figure S4. *infB* gene-gene network. *infB* is shown by an arrow.

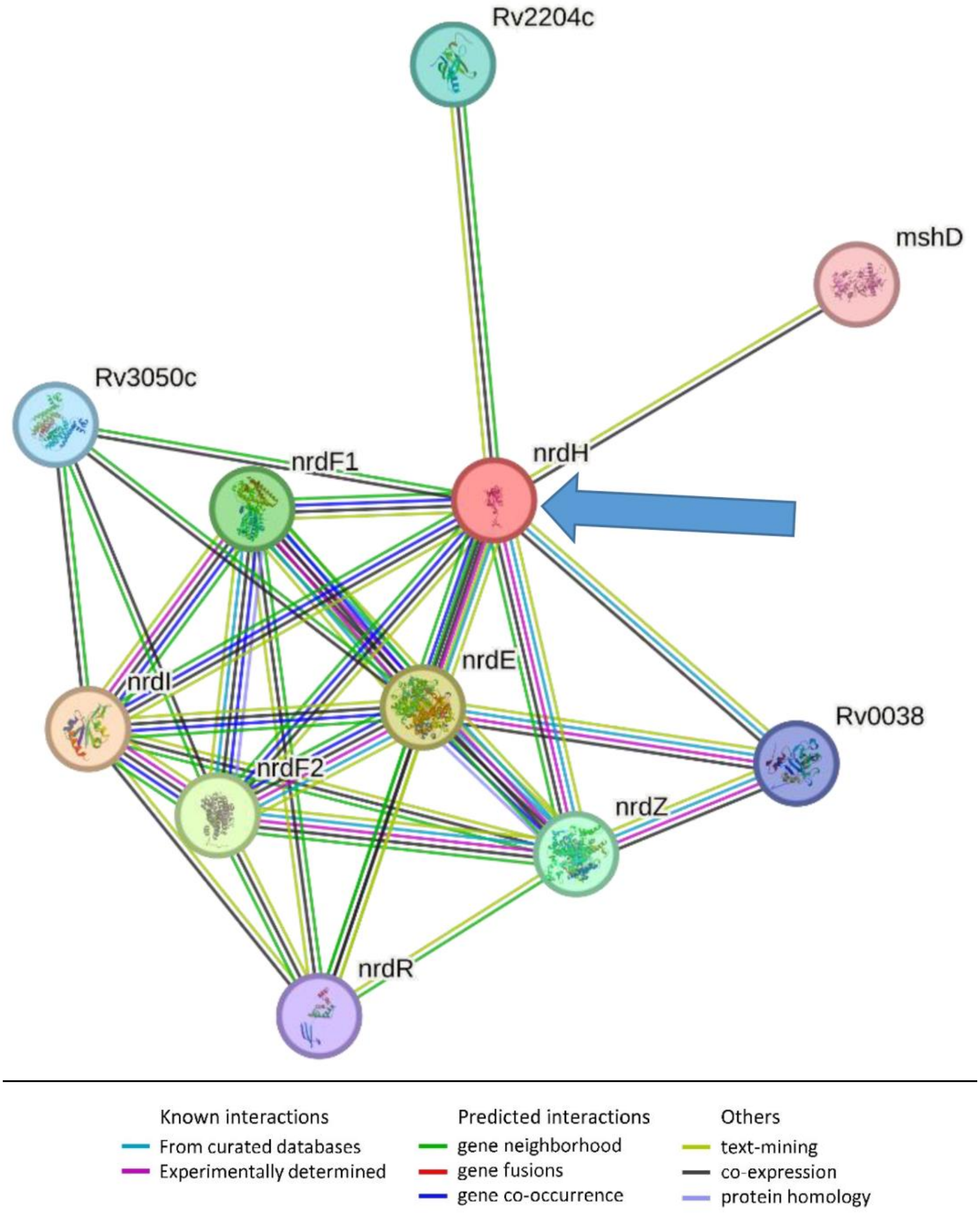

Figure S5. *nrdH* gene-gene network**.** *nrdH* is shown by an arrow.
