# Supplementary material for: Genomic Insight into Primary Adaptation of Mycobacterium tuberculosis to Aroylhydrazones and Nitrofuroylamides In Vitro
Source: Antibiotics (Basel). 2025 Feb 22;14(3):225. doi: 10.3390/antibiotics14030225 (PMC11939388; doi:10.3390/antibiotics14030225)
Supplement: Supplementary file 1 [file antibiotics-14-00225-s001.zip › Table S1. Clonal variants of M. tuberculosis H37Rv.pdf]

Genomic Insight into Primary Adaptation of *Mycobacterium tuberculosis* to Aroylhydrazones and Nitrofuroylamides *in vitro*

Igor Mokrousov, Violina T. Angelova, Ivaylo Slavchev, Mikhail Bezruchko, Simeon Dimitrov, Dmitrii E. Polev, Georgi M. Dobrikov, Violeta Valcheva

Supplementary Table S1. Clonal variants with mutations derived from the parental strain *M. tuberculosis* H37Rv grown on solid media with increased concentrations of different compounds.

Sample	Compound, concentration, in µg/ml and relative to MIC	Gene label and name, mutation, position in gene and genome, comment
1bgm	VAL_SNN<br>2.56 µg/ml (10x MIC)	*Rv3696c / glpK*. Genome position 4139183 A>AC insert_frameshift within CCCCCC > CCCCCCC. Gene position 573. Frameshift in codon 191 (all protein is 517 aa).<br>*Rv3366 / spoU*. Genome position 3777769 C>T. Gene position: 33 C>T. Synonymous mutation in *Rv3366* codon 11 I>I, ATC>ATT.
3bgm	MLT_FUR<br>0.28 µg/ml (4x MIC)	*Rv3755c.* Genome position 4201587 T>C. Gene position 302 A>G. Nonsynonymous mutation in *Rv3755c* codon 101 H>R, CAC>CGC.
Bgm8-24	MLT_FUR<br>0.56 µg/ml (8x MIC)	*Rv3696c / glpK*. Genome position 4139183 A>AC insert_frameshift within CCCCCC > CCCCCCC. Gene position 573. Frameshift in codon 191 (all protein is 517 aa).<br>*Rv3366 / spoU*. Genome position 3777769 C>T. Gene position: 33 C>T. Synonymous mutation in *Rv3366* codon 11 I>I, ATC>ATT.
Bgm9-24	MLT_FUR<br>0.56 µg/ml (8x MIC)	*Rv3755c.* Genome position 4201587 T>C. Gene position 302 A>G. Nonsynonymous mutation in *Rv3755c* codon 101 H>R, CAC>CGC.
Bgm10-2024	MLT_FUR<br>0.28 µg/ml (4x MIC)	*Rv2702 / ppgK.* Genome position 3017408; Delete_frameshift del_C. Gene position 551. This frameshift occurs in codon 184 (all protein is 265 aa).<br>*Rv0506 / mmpS2.* Genome position 597110 A>G. Gene position 352. Nonsynonymous mutation in *Rv0506* codon 118 S>G, AGC>GGC.
Bgm3-2024	DO-209<br>0.16 µg/ml (8x MIC)	*Rv3053c / nrdH.* Genome position: 3415181, deletion del_CACCTAGGGGGTGG at position -223 upstream of start codon.<br>*Rv2839c / infB.* Genome position: 3147075 A>C. Gene position: 799. Nonsynonymous mutation in *Rv2839c* codon 267 F>V, TTC>GTC.

Note. Mutations were detected by SAM-TB tool and confirmed by using Geneious R package.
